# RUNX1-IT1 favors breast cancer carcinogenesis through regulation of IGF2BP1/GPX4 axis

**DOI:** 10.1007/s12672-023-00652-z

**Published:** 2023-04-10

**Authors:** Shengting Wang, Yufang Wang, Qian Li, Kaixuan Zeng, Xiaoming Li, Xinghua Feng

**Affiliations:** 1 Clinical Medical Center, Xi’an Peihua University, 888 Changning Street, Xi’an, 710125 Shaanxi China; 2 School of Medicine, Xi’an Jiaotong University, Xi’an, 710000 China

**Keywords:** Long non-coding RNA, Breast cancer, Ferroptosis, m^6^A reader, mRNA stability

## Abstract

Breast cancer is the most common malignancy among women and the leading cause of cancer deaths, with complicated pathogenesis that is largely unknown. In this study, we identified a novel long non-coding RNA (lncRNA) as a critical driver of breast cancer tumorigenesis. RUNX1 intronic transcript 1 (RUNX1-IT1) was notably overexpressed in human breast cancer tissues, and knockdown of RUNX1-IT1 inhibited breast cancer cell viability and invasion, as well as tumor growth in orthotopic transplantation model. Further, RUNX1-IT1 repressed ferroptosis, a novel iron-dependent form of regulated cell death, via increasing glutathione peroxidase 4 (GPX4) expression. Specifically, RUNX1-IT1 directly bound to N6-methyladenosine m^6^A reader IGF2BP1 and promoted the formation of (insulin like growth factor 2 mRNA binding protein 1) IGF2BP1 liquid-liquid phase separation (LLPS) biomolecular condensates, resulting in more IGF2BP1 occupation on GPX4 mRNA, increasing GPX4 mRNA stability. Moreover, high RUNX1-IT1 was linked to poor prognosis, and a strong positive correlation between RUNX1-IT1 and GPX4 was observed in clinical breast cancer tissues. Taken together, our data reveal that RUNX1-IT1 promotes breast cancer carcinogenesis through blocking ferroptosis via elevating GPX4, targeting of the previously unappreciated regulatory axis of RUNX1-IT1/IGF2BP1/GPX4 may be a promising treatment for patient with breast cancer.

**Supplementary Information:**

The online version contains supplementary material available at 10.1007/s12672-023-00652-z.

## Introduction

Breast cancer is a phenomenon of uncontrolled proliferation of breast epithelial cells under the action of a variety of carcinogens [1]. In the early stage, the disease is often manifested as breast mass, nipple discharge, axillary lymph node enlargement and other symptoms; while in the late stage, cancer cells may metastasize in the distance and appear multiple organ lesions, which directly threaten the life of patients [2, 3]. The pathogenesis of breast cancer is complex, and the exact cause of breast cancer is still poorly understood. In-depth study of the potential mechanism of breast cancer occurrence and development may uncover new therapeutic targets and bring new ideas for clinical treatment.

More than 90% of the transcripts of the human genome are non-coding RNA, which participate in the regulation of gene expression at different levels, not only controlling the basic biological processes such as growth and development, organ function, but also playing an important role in the whole human disease [4]. Long non-coding RNA (lncRNA) is a non-coding transcript with a length of more than 200 nucleotides transcribed by an independent promoter [5]. At first, it was considered as a non-functional transcription “noise”, and later proved to play an important regulatory role in a variety of physiological and pathological processes [6]. Accumulated evidence shows that lncRNA is indispensable for tumor initiation, development and progression [7]. To date, some lncRNAs have been reported to be associated with breast cancer, for instance, AGAP2-AS1 (AGAP2 antisense RNA 1) promoted breast cancer stemness and trastuzumab resistance by controlling choline phosphotransferase 1-meidiated fatty acid oxidation [8]. TOMM22 divergent transcript (Lnc-408) was proposed as an inducer of epithelial-mesenchymal transformation, and promoted breast cancer cell invasion and metastasis by regulating cytoskeletal stability [9]. And breast cancer-related TINCR ubiquitin domain containing (TINCR) impaired the efficacy of immunotherapy via posttranslational modification of programmed cell death 1 ligand 1 (PDL1) [10]. These studies highlight the importance of lncRNA in breast cancer.

Ferroptosis is a new type of iron-dependent programmed cell death, which is different from apoptosis, necrosis and autophagy [11]. The occurrence of ferroptosis is due to the accumulation of reactive oxygen species (ROS) and the depletion of reducing glutathione (GSH), leading to the oxidation of iron-dependent polyunsaturated fatty acid (PUFA), eventually destroying the plasma membrane structure and resulting in cell death [12]. Emerging evidence suggests that ferroptosis is an obstacle to cancer progression [13]. In order to resist excessive ROS produced by rapid proliferation, tumor cells will adaptively improve their antioxidant capacity, hence, interference with the redox balance in tumor cells is likely to lead to ferroptosis and achieve the goal of treating tumor [14]. Glutathione peroxidase 4 (GPX4), a unique antioxidant enzyme, can reduce lipid hydroperoxides to lipid alcohols and prevent ROS accumulation in the body, has been proven to be an essential inhibitor of ferroptosis [15]. GPX4 is highly expressed in human pan-carcinoma tissues and negatively correlated with survival time [16]. When GPX4 is inhibited by drugs, cancer cells lose the antioxidant enzymes that specifically scavenge lipid peroxides, resulting in excessive accumulation of lipid peroxides and ferroptosis; meanwhile, GPX4 inhibition also cause the inactivation of GSH, making cancer cells more vulnerable to oxidative damage [17].

In the present study, by analyzing lnCAR database (https://lncar.renlab.org/) that integrates human lncRNA expression profiles spanning diverse cancers from Gene Expression Omnibus (GEO) database, we found that some lncRNAs were deregulated in breast cancer. The top five upregulated lncRNAs were long intergenic non-protein coding RNA 1614 (LINC01614), solute carrier family 20 member 1 (SLC20A1), DSCAM antisense RNA 1 (DSCAM-AS1), HOX transcript antisense RNA (HOTAIR) and RUNX1-IT1, of which only RUNX1-IT1 has not been previously characterized in breast cancer. Recently, RUNX1-IT1 has been proposed as an oncogenic lncRNA in several cancers, such as lung cancer [18], glioblastoma [19] and pancreatic cancer [20]. Conversely, RUNX1-IT1 was identified as a tumor suppressor in gastric cancer [21] and hepatocellular carcinoma [22]. These studies demonstrate that RUNX1-IT1 functions in a context-dependent manner. Herein, we aimed to explore the role, function, potential regulatory network and clinical significance of RUNX1-IT1 in breast cancer.

## Materials and methods

In this study, we first tested the expression of RUNX1-IT1 in normal and breast cancer tissues, and analyzed the correlations between RUNX1-IT1 and clinical features of breast cancer patients. Then, we analyzed the functions of RUNX1-IT1 by knockdown of RUNX1-IT1 in T47D and MDA-MB-231 cells, followed by Cell Counting Kit-8 (CCK-8), Transwell and ferroptosis detection assays. Next, the mechanism of RUNX1-IT1 was analyzed by RNA pull-down, immunoprecipitation, western blot and phase separation assays in T47D and MDA-MB-231 cells. Lastly, the in vivo function of RUNX1-IT1 was analyzed by using breast cancer orthotopic transplantation model.

### Tissue samples

The protocol of sample collection and use was approved by the Ethics Committee of The Affiliated Shaanxi Fourth People Hospital of Peihua University, which was conducted in compliance with the guidelines of the Declaration of Helsinki. 70 paired breast cancer and adjacent normal samples were fresh tissues removed surgically, which were immediately put into liquid nitrogen to avoid RNA degradation. Patients were routinely followed up every three months after discharge. The detailed clinicopathological information of all patients is included in Table 1.

**Table 1:** Association of RUNX1-IT1 expression with the clinical features in breast cancer patients (n = 70)

Parameters	Total (n = 70)	RUNX1-IT1 expression		*P* value
		Low (n = 35)	High (n = 35)	
Age (years)				
≤ 40	18	11	7	0.274
> 40	52	24	28	
Tumor size (cm)				
≤ 2	44	28	16	0.003
> 2	26	7	19	
TNM stage				
I-II	41	27	14	0.002
III-IV	29	8	21	
ER status				
Negative	28	13	15	0.626
Positive	42	22	20	
PR status				
Negative	31	21	10	0.099
Positive	39	24	25	
HER2 status				
Negative	55	29	26	0.382
Positive	15	6	9	
TNBC				
Yes	13	8	5	0.356
No	57	27	30	

*LN* lymph node; *ER* estrogen receptor, *PR* progesterone receptor, *HER2* human epidermal growth factor receptor 2 ; *TNBC* triple-negative breast cancer;

### Reagents

Erastin (a ferroptosis inducer) and Ferrostatin-1 (a ferroptosis inhibitor) were obtained from Selleck Chemicals (Houston, TX, USA). ZVAD-FMK (an apoptosis inhibitor) and necrosulfonamide (a necroptosis inhibitor) were purchased from Sigma (St. Louis, MO, USA).

### Cell lines, constructs and transfection

Two breast cancer cell lines T47D and MDA-MB-231 were purchased from ATCC, and cultured in DMEM medium. To stably knock down RUNX1-IT1, two shRNAs targeting RUNX1-IT1 (#1: 5′-TCGAAGACATCGGCAGAAA-3′; #2: ACCACTCCACTGCCTTTAA; Negative control (NC): TTCTCCGAACGTGTCACGT) were inserted into pLKO.1-puro lentiviral vector, followed by infection into T47D and MDA-MB-231 cells. The stable clone was screened by puromycin for 1 week. GPX4 vector was purchased from OriGene (#RC208065) and transfected into T47D and MDA-MB-231 cells to overexpress GPX4. Besides, the full-length coding sequences (CDS) of IGF2BP1 and deletion of KH3/4 mutant were cloned into pEGFP-N1 vector, followed by transfection into HEK293T cells to overexpress IGF2BP1 and its mutant. Protein affinity purification was conducted by using Anti-GFP Magnetic Beads (#AE079, ABclonal, MA, USA) as per the manufacturer’s instructions. Cell transfection was carried out using Lipofectamine 3000 (Thermo Fisher Scientific, CA, USA).

### Quantitative real-time PCR

Total RNA was extracted using RNeasy Plus Universal Kits (Qiagen, Dusseldorf, Germany), followed by quantification using NanoDrop ND-2000 spectrophotometer. Reverse transcription and real-time PCR was performed using a PrimeScript RT reagent and SYBR^®^ Premix Ex Taq™ kits (Takara, Otsu, Japan), respectively, according to the manufacturer’s protocol. Gene expression was normalized to GAPDH expression using 2^−ΔΔCt^ method.

### CCK-8 and transwell assays

T47D and MDA-MB-231 cells with stable RUNX1-IT1 knockdown were plated onto 96-well plates, 10 µL CCK-8 solution (MedChemExpress, NJ, USA) was added into cell medium and incubated for 2 h. The light absorption value at 450 nm was recorded. Cell invasion was conducted using 8 μm Transwell chamber coated with matrigel (Corning, MA, USA).

### Animal study

MDA-MB-231 cells with stable RUNX1-IT1 knockdown were injected into NOD/SCID mice, which were randomly divided into three groups, five in each group. The protocol of establishment of breast cancer orthotopic transplantation model was described in our previous study [23]. All animal studies were performed following institutional guidelines of the Animal Care and Use Committee of Peihua university. Extensive efforts were made to ensure minimal suffering of the mice used in this study.

### Western blot

Cells were lysed in cold, freshly prepared radio immunoprecipitation assay (RIPA) buffer (Sigma, MO, USA) and quantified with a Pierce™ BCA Protein Assay Kit (Thermo Fisher Scientific). 30 µg total protein samples were separated by gradient SDS-polyacrylamide gel and transferred to polyvinylidene fluoride (PVDF, Millipore, USA) membranes for immunoblotting. The primary antibodies used in this study: anti-PCNA (#13,110, Cell Signaling Technology), anti-IGF2BP1 (#8482, Cell Signaling Technology) and anti-GAPDH (TA802519, OriGene).

### Detection of ferroptosis

The free Fe^2+^ were assessed using Iron Assay Kit (#ab83366, Abcam), and the content of GSH was analyzed using GSH/GSSG Ratio Detection Assay (#ab138881, Abcam). Malondialdehyde (MDA) Assay Kit (competitive ELISA) (#ab238537, Abcam) was used for the rapid detection and quantitation of MDA-protein adducts. And lipid ROS was tested by Image-iT™ Lipid Peroxidation Kit (#C10445, Thermo Fisher Scientific) according to the manufacturer’s instructions. Cell death was detected by PI staining.

### RNA pull-down and immunoprecipitation (RIP)

The full-length of RUNX1-IT1 was in vitro synthesized and labeled with biotin (Sangon, Shanghai, China), followed by incubated with T47D and MDA-MB-231 cell lysates for 3 h. Then, the Streptavidin Magnetic Beads (#88,817, Thermo Fisher Scientific) were added and incubated for 0.5 h. The pull-down proteins were analyzed by western blot and mass spectrum. Mass spectrum was carried out using a Q-Exactive mass spectrometer (Introvigen). In brief, the pull-down proteins were loaded onto the C18-reversedphase analytical column, followed by separation by a linear gradient of buffer containing 80% acetonitrile and 0.1% formic acid. Then, the samples were subjected to mass spectrometer analysis for 1 h in a positive ion mode. Dynamic exclusion for selected ions was 30s, and the normalized collision energy was 27 eV. The protocol of RIP assay was described in Magna RIP™ RNA-Binding Protein Immunoprecipitation Kit (#17–700, Sigma).

### In vitro phase separation

The purified GFP-IGF2BP1 protein and synthesized Cy-3 labeled RUNX1-IT1 probe were added into a physiological liquid-liquid phase separation (LLPS) buffer (20 mM Tris-HCl, pH 7.5, 15 mM NaCl, 130 mM KCl, 5 mM KH_2_PO_4_, 1.5 mM MgCl_2_, and 1 mg/mL BSA) with 10% PEG8000. The results were observed on glass-bottomed dishes with a confocal microscopy.

### Fluorescence recovery after photobleaching (FRAP) assay

FRAP assay was used to measure the fluidity of IGF2BP1 liquid droplets, which is an unique characteristic of protein LLPS. The purified wild-type and mutant GFP-IGF2BP1 proteins were added onto glass-bottomed dishes, followed by bleaching in a circular region of interest with 100% laser power using 488-nm lasers. Time-lapse images after bleaching were recorded at the indicated time.

### ***In silico*** analysis

The differentially expressed lncRNAs in breast cancer were analyzed by using lnCAR online tool (https://lncar.renlab.org/) that integrates human lncRNA expression profiles spanning diverse cancers from GEO database. And RUNX1-IT1 expression in normal and breast cancer tissues from The Cancer Genome Atlas (TCGA) database was analyzed by using UALCAN online tool (https://ualcan.path.uab.edu/).

### Statistical analysis

The differential expression of RUNX1-IT1 in breast cancer tissues was tested by paired Student’s t-test. Multi-group differences were compared using ANOVA. Kaplan-Meier plotter was used to analyze the effect of RUNX1-IT1 on the overall survival time of breast cancer patients, which was tested by log-rank test. The correlations between RUNX1-IT1 and clinicopathological features of patients were determined by Chi-square test. Data were shown as mean ± standard error, figures were plotted by using Graphpad prism software. All statistical models are two-tailed. *P < 0.05, **P < 0.01, **P < 0.001.

## Results

### RUNX1-IT1 is overexpressed in breast cancer

**Fig. 1:**
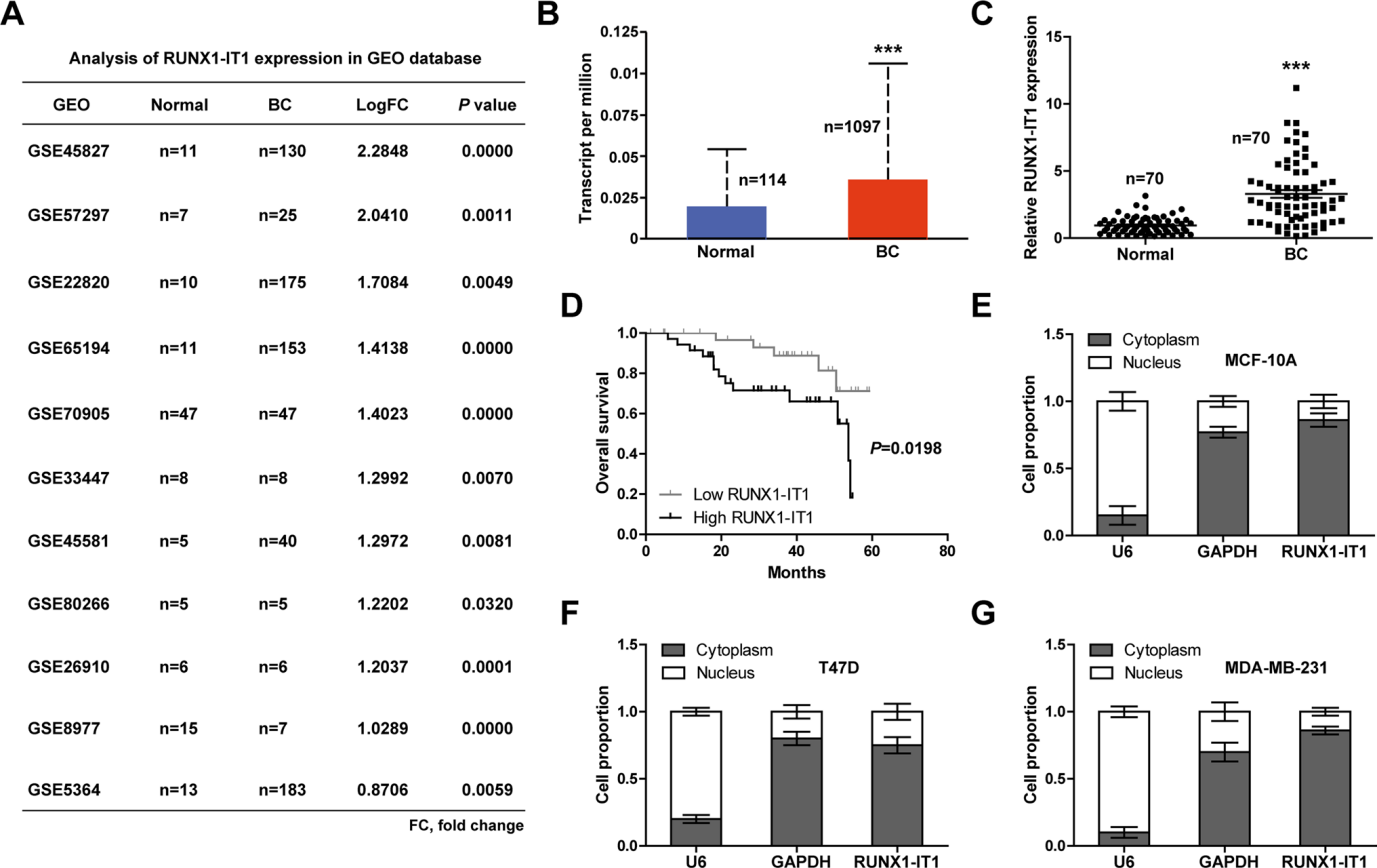
Identification of RUNX1-IT1 as a upregulated lncRNA in breast cancer. **A**, **B** Analysis of RUNX1-IT1 expression in breast cancer in GEO and TCGA databases. **C** qRT-PCR analysis of RUNX1-IT1 expression in paired normal and breast cancer tissues. **D** The survival curve of breast cancer patients based on RUNX1-IT1 expression levels. **E**–**G** qRT-PCR analysis of the location of RUNX1-IT1 in the indicated cells. ***P < 0.001

Through analyzing 11 GEO profiles containing lncRNA differential expression between breast cancer and normal tissues, we found that RUNX1-IT1 was significantly upregulated in breast cancer tissues (Fig. 1A). Moreover, TCGA database showed that breast cancer tissues had higher RUNX1-IT1 levels than normal tissues (Fig. 1B). Then, we tested RUNX1-IT1 expression in our cohort (Fig. 1C), the results were consistent with GEO and TCGA databases. High RUNX1-IT1 was significantly correlated with larger tumor volume and later clinical stage (Table 1). Further, breast cancer patients with high RUNX1-IT1 displayed poor outcome, as shown by Kaplan-Meier survival curve (Fig. 1D). The results of qRT-PCR showed that RUNX1-IT1 was predominantly located in the cytoplasm in both normal and breast cancer cell lines (Fig. 1E–G). These data showed that RUNX1-IT1 is a cytoplasmic lncRNA that may be responsible for breast cancer progression.

### Knockdown of RUNX1-IT1 inhibits breast cancer malignant behavior

**Fig. 2:**
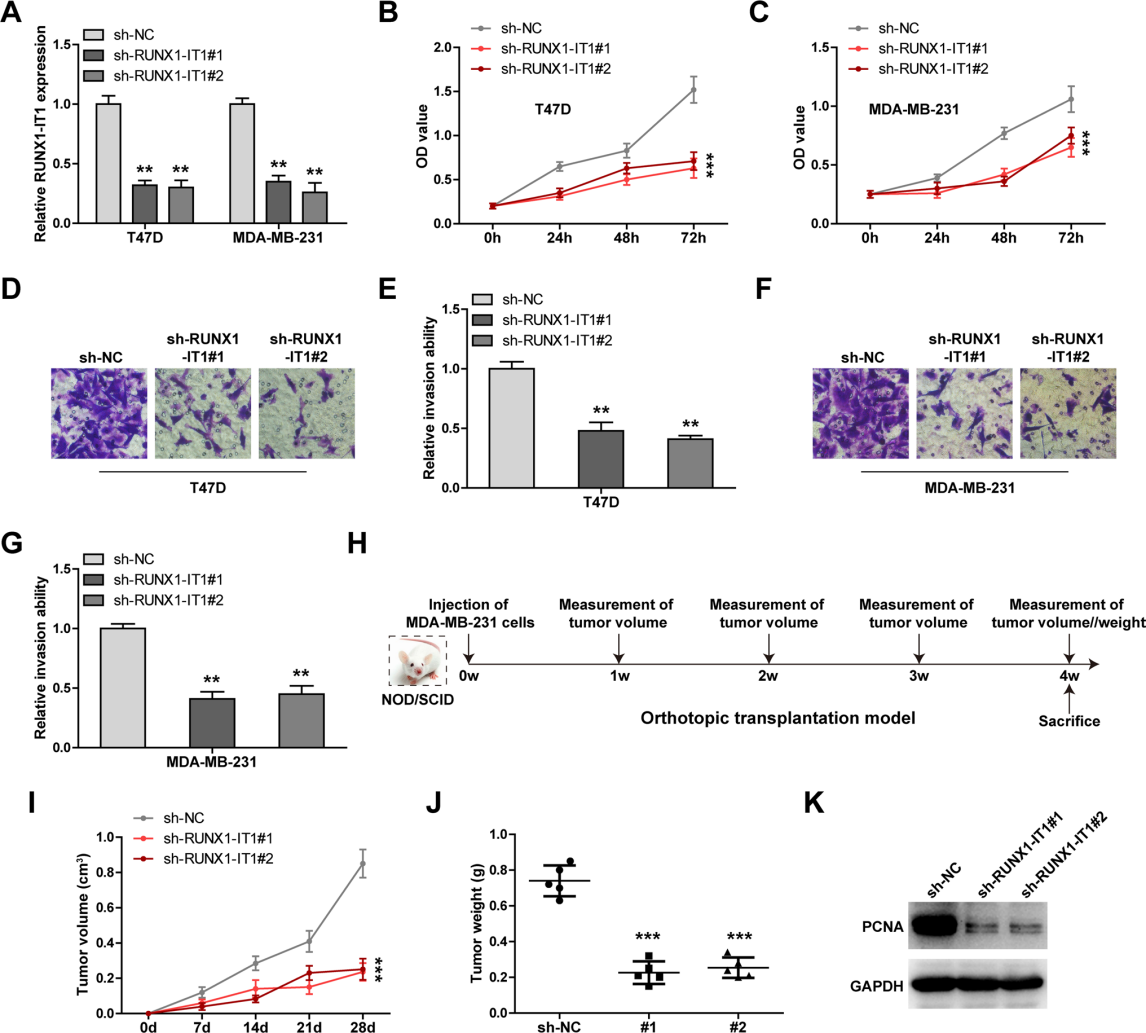
Identification of RUNX1-IT1 function in breast cancer. **A** qRT-PCR verifying the knockdown efficiency of RUNX1-IT1. **B**, **C** CCK-8 assay testing cell viability in cells with RUNX1-IT1 silencing. **D**–**G** Transwell assay testing cell invasion after RUNX1-IT1 silencing. **H** The flowchart showing the protocol of establishment of orthotopic transplantation model. I, J. Tumor volume and weight of mice in the indicated groups. **K** Western blot testing PCNA levels in the indicated groups. GAPDH was used the loading control. **P < 0.01, ***P < 0.001

We established stable RUNX1-IT1-silenced breast cancer cell lines (Fig. 2A), and performed a series of functional assays. The results of CCK-8 assay showed that silencing of RUNX1-IT1 inhibited cell viability at the indicated time point (Fig. 2B, C). And RUNX1-IT1-silenced T47D and MDA-MB-231 cells displayed weaker invasiveness than control cells (Fig. 2D–G). To verify the function of RUNX1-IT1 in vivo, we established orthotopic transplantation model by injecting cells into the abdominal mammary fat pad of NOD/SCID mice (Fig. 2H). After four weeks, mice were euthanized and the results showed that tumor volume and weight were reduced after depletion of RUNX1-IT1 (Fig. 2I, J). Consistently, less PCNA (a proliferation marker) levels were observed in RUNX1-IT1-silenced group as compared to control group (Fig. 2K).

### RUNX1-IT1 regulates ferroptosis in breast cancer

**Fig. 3:**
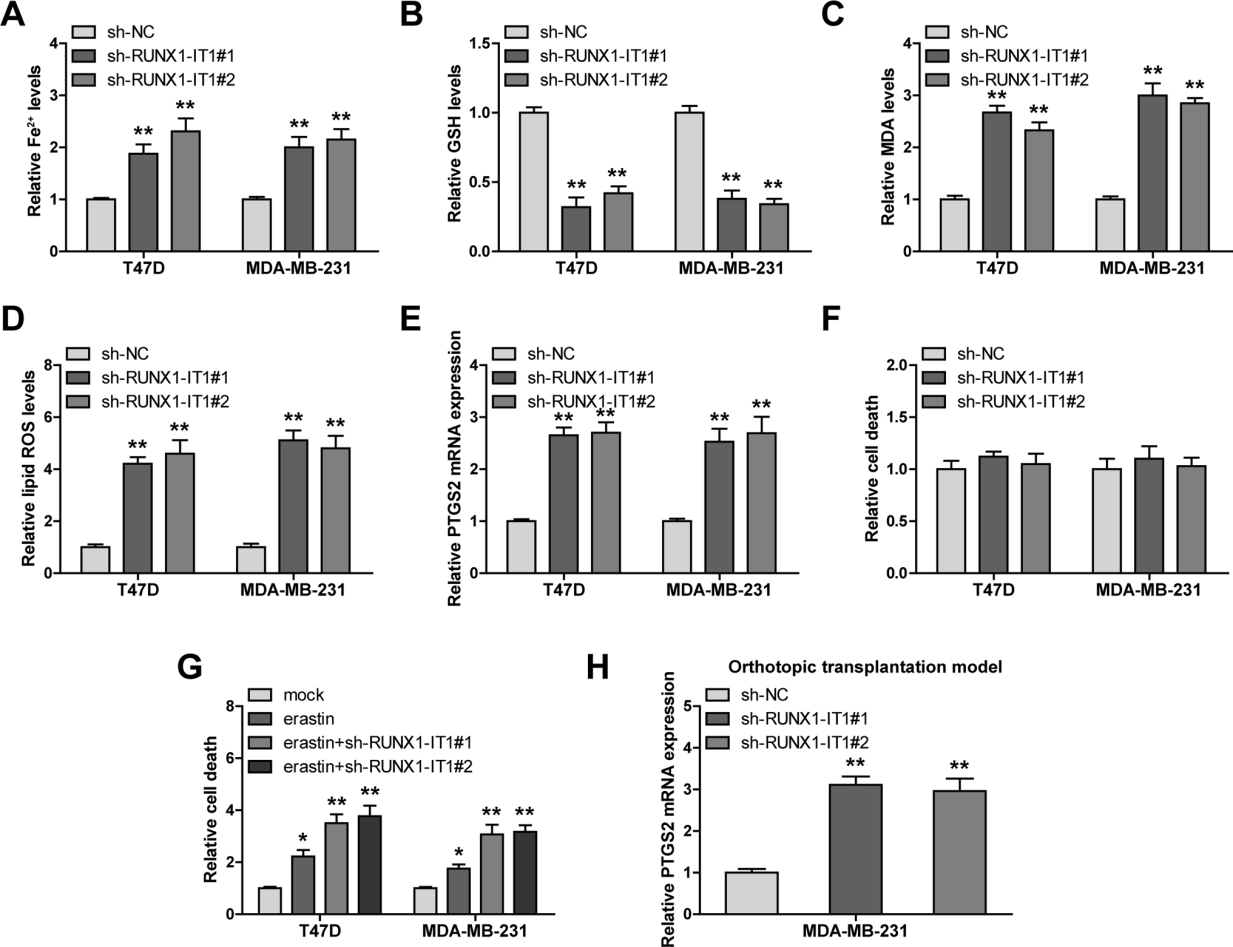
RUNX1-IT1 inhibits ferroptosis. **A**–**D** The levels of Fe^2+^, GSH, MDA and lipid ROS were tested after RUNX1-IT1 knockdown. **E** qRT-PCR analysis of PTGS2 mRNA levels in RUNX1-IT1-silenced cells. **F**, **G** PI staining testing cell death in RUNX1-IT1-silenced cells. **H** qRT-PCR analysis of PTGS2 mRNA levels in tumors bearing RUNX1-IT1-silenced MDA-MB-231 cells. *P < 0.05, **P < 0.01

As shown in Fig. 3A, the content of Fe^2+^ in T47D and MDA-MB-231 cells was significantly increased by RUNX1-IT1 knockdown, along with decreased GSH levels (Fig. 3B) but increased MDA (Fig. 3C) and lipid ROS (Fig. 3D) levels. Moreover, silencing of RUNX1-IT1 notably increased expression of PTGS2 (Fig. 3E), a ferroptosis marker. Further, RUNX1-IT1 knockdown did not affect T47D and MDA-MB-231 cell death (Fig. 3F), whereas enhanced erastin (a ferroptosis inducer)-induced death (Fig. 3G). And the increased ferroptotic responses caused by RUNX1-IT1 knockdown were significantly blocked by Ferrostatin-1 (a ferroptosis inhibitor), but not by ZVAD-FMK (an apoptosis inhibitor) and necrosulfonamide (a necroptosis inhibitor) (Fig. S1A, B). Likewise, the in vivo model also showed that RUNX1-IT1 was a negative regulator of ferroptosis marker PTGS2 (Fig. 3H). These data suggest that knockdown of RUNX1-IT1 induces ferroptosis.

### RUNX1-IT1 blocks ferroptosis by elevating GPX4

**Fig. 4:**
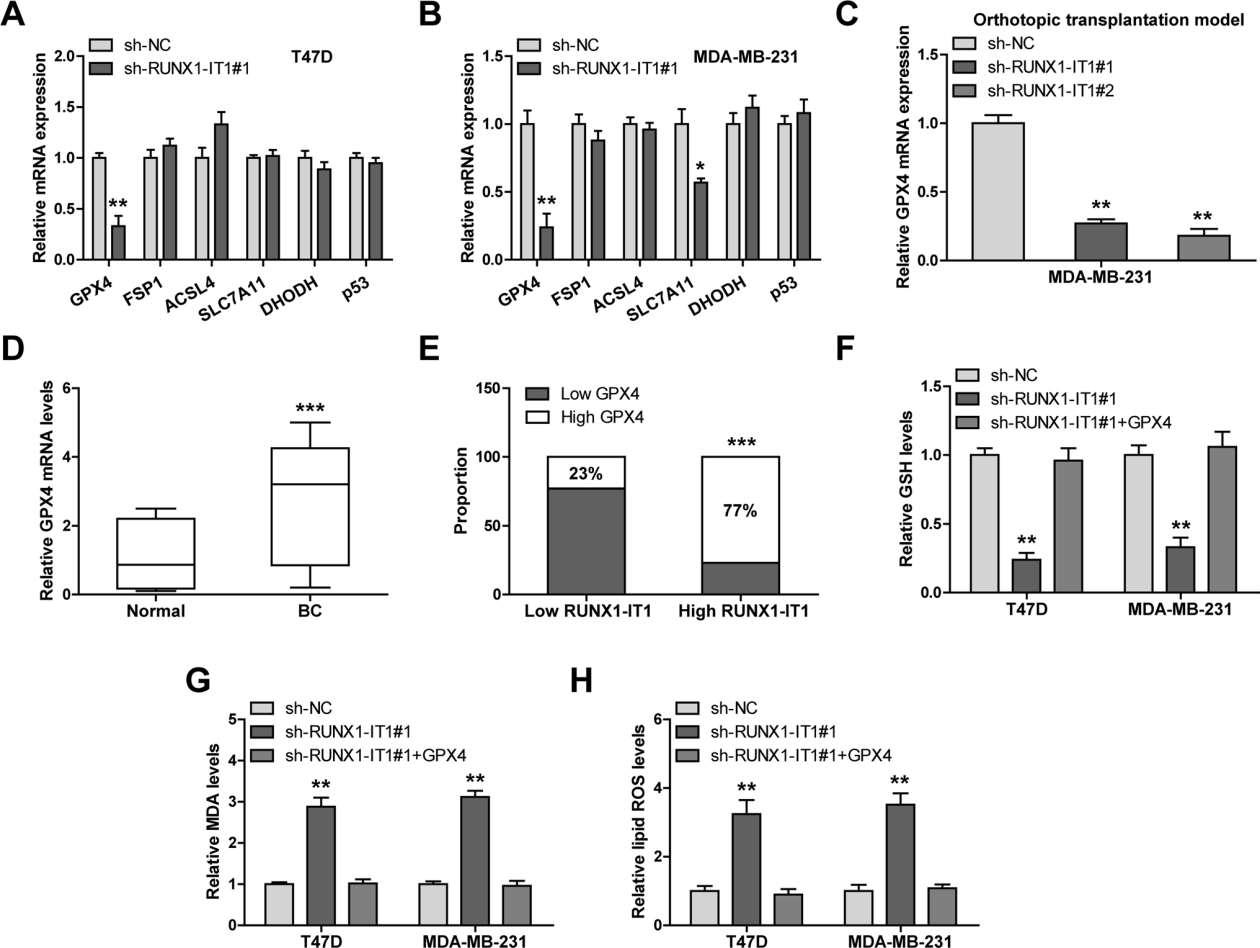
RUNX1-IT1 regulates GPX4 expression. **A**, **B** qRT-PCR analysis of the indicated gene expression in RUNX1-IT1-silenced cells. **C**, **D** qRT-PCR analysis of GPX4 expression in mice tumor tissues and human breast cancer tissues. **E** The correlation between RUNX1-IT1 and GPX4 expression in breast cancer tissues. **F**–**H** Detection of GSH, MDA and lipid ROS levels in RUNX1-IT1-silenced cells transfected with GPX4 vector. *P < 0.05, **P < 0.01, ***P < 0.001

To explore how RUNX1-IT1 affected ferroptosis, we tested the expression of ferroptosis-related genes, and found that only GPX4 was consistently downregulated in both T47D and MDA-MB-231 cells (Fig. 4A, B). Low GPX4 was also observed in tumor bearing RUNX1-IT1-silenced MDA-MB-231 cells (Fig. 4C). In addition, GPX4 was significantly overexpressed in breast cancer tissues in comparison to normal tissues (Fig. 4D), and its expression was strongly positively correlated with RUNX1-IT1 expression (Fig. 4E). The reduced GSH and increased MDA and lipid ROS caused by RUNX1-IT1 silencing were almost completely counteracted by GPX4 overexpression in both T47D and MDA-MB-231 cells (Fig. 4F–H). These data indicate that GPX4 is a downstream target of RUNX1-IT1.

### RUNX1-IT1 regulates GPX4 mRNA stability via IGF2BP1

**Fig. 5:**
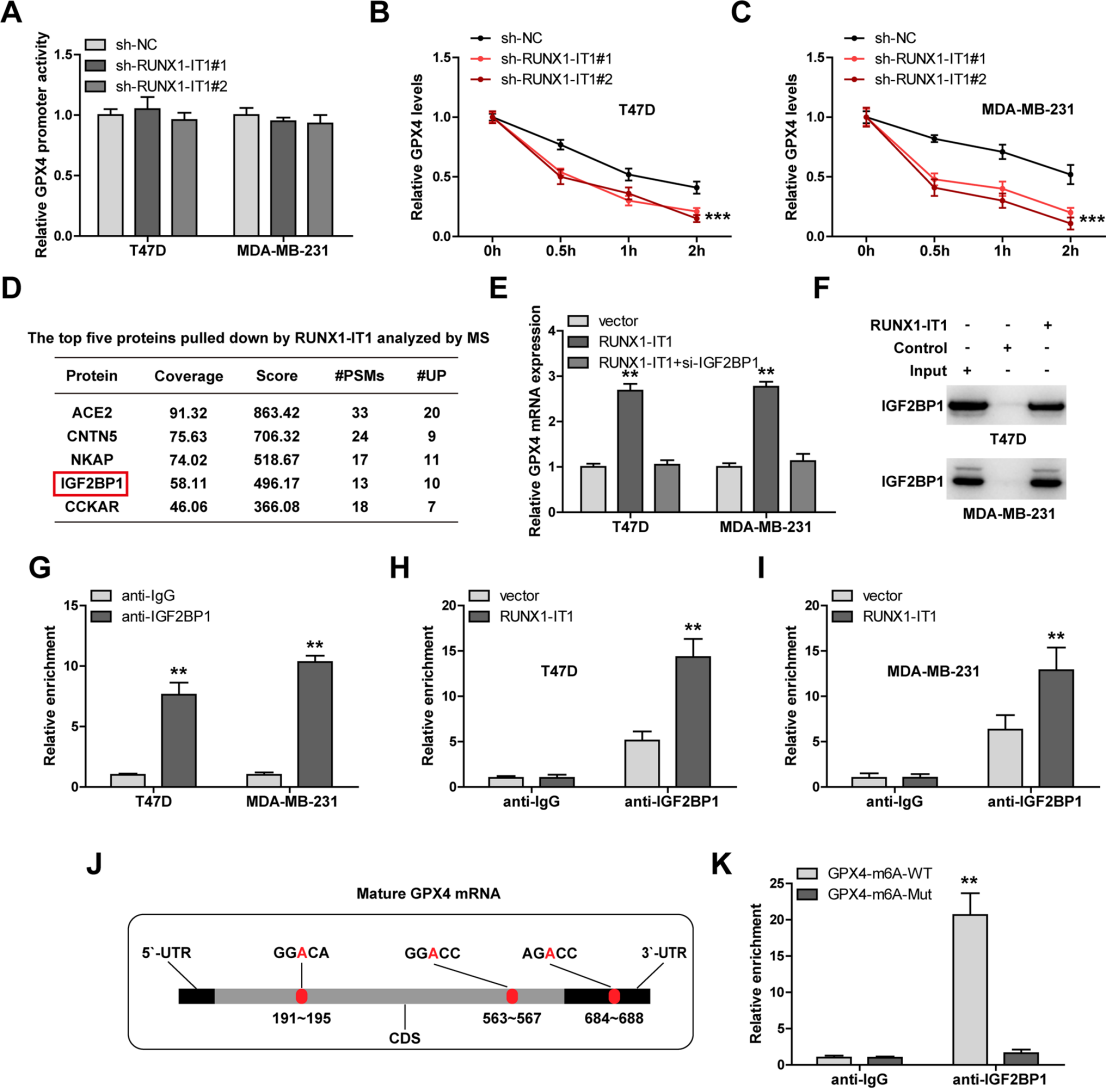
RUNX1-IT1 regulates GPX4 via binding to IGF2BP1. **A** Luciferase reporter assay testing GPX4 promoter activity affected by RUNX1-IT1. **B**, **C** qRT-PCR analysis testing the half-life of GPX4 mRNA in RUNX1-IT1-silenced cells treated with 10µM actinomycin D at the indicated time. **D** RNA pull-down assay using RUNX1-IT1 probe, followed by mass spectrometry (MS). **E** qRT-PCR analysis of GPX4 expression in RUNX1-IT1-overexpressed cells transfected with IGF2BP1 siRNA. **F**, **G** RNA pull-down and RIP assays testing the interaction between RUNX1-IT1 and IGF2BP1. **H**, **I** RIP assay testing the binding of IGF2BP1 to GPX4 mRNA in RUNX1-IT1-overexpressed cells. **J** Three m^6^A sites were found on mature GPX4 mRNA. **K** RIP assay testing the binding of IGF2BP1 to GPX4 mRNA with or without m^6^A mutation. **P < 0.01

As shown in Fig. 5A, RUNX1-IT1 did not affect GPX4 promoter activity, but the half-life of GPX4 mRNA was evidently shortened after RUNX1-IT1 knockdown (Fig. 5B, C). To understand how RUNX1-IT1 controlled GPX4 mRNA stability, we performed RNA pull-down coupled mass spectrometric analysis, the results showed that m^6^A reader IGF2BP1, a mRNA stabilizer, was pulled down by RUNX1-IT1 (Fig. 5D). Knockdown of IGF2BP1 significantly reduced the half-life of GPX4 mRNA (Fig. S2A, B), and blocked the elevated GPX4 expression caused by RUNX1-IT1 overexpression (Fig. 5E), hinting that IGF2BP1 is involved in RUNX1-IT1-mediated regulation of GPX4. RNA pull-down coupled western blot and RIP assays showed that RUNX1-IT1 and IGF2BP1 interacted with each other (Fig. 5F, G). Moreover, overexpression of RUNX1-IT1 increased the binding of IGF2BP1 to GPX4 mRNA in both T47D and MDA-MB-231 cells (Fig. 5H, I). Mature GPX4 mRNA has three m^6^A binding motifs (Fig. 5J), mutation of these sites entirely blocked the interaction between IGF2BP1 and GPX4 mRNA (Fig. 5K). These results suggest that RUNX1-IT1 increases GPX4 mRNA stability via binding to IGF2BP1.

### RUNX1-IT1 drives IGF2BP1 liquid droplet formation

**Fig. 6:**
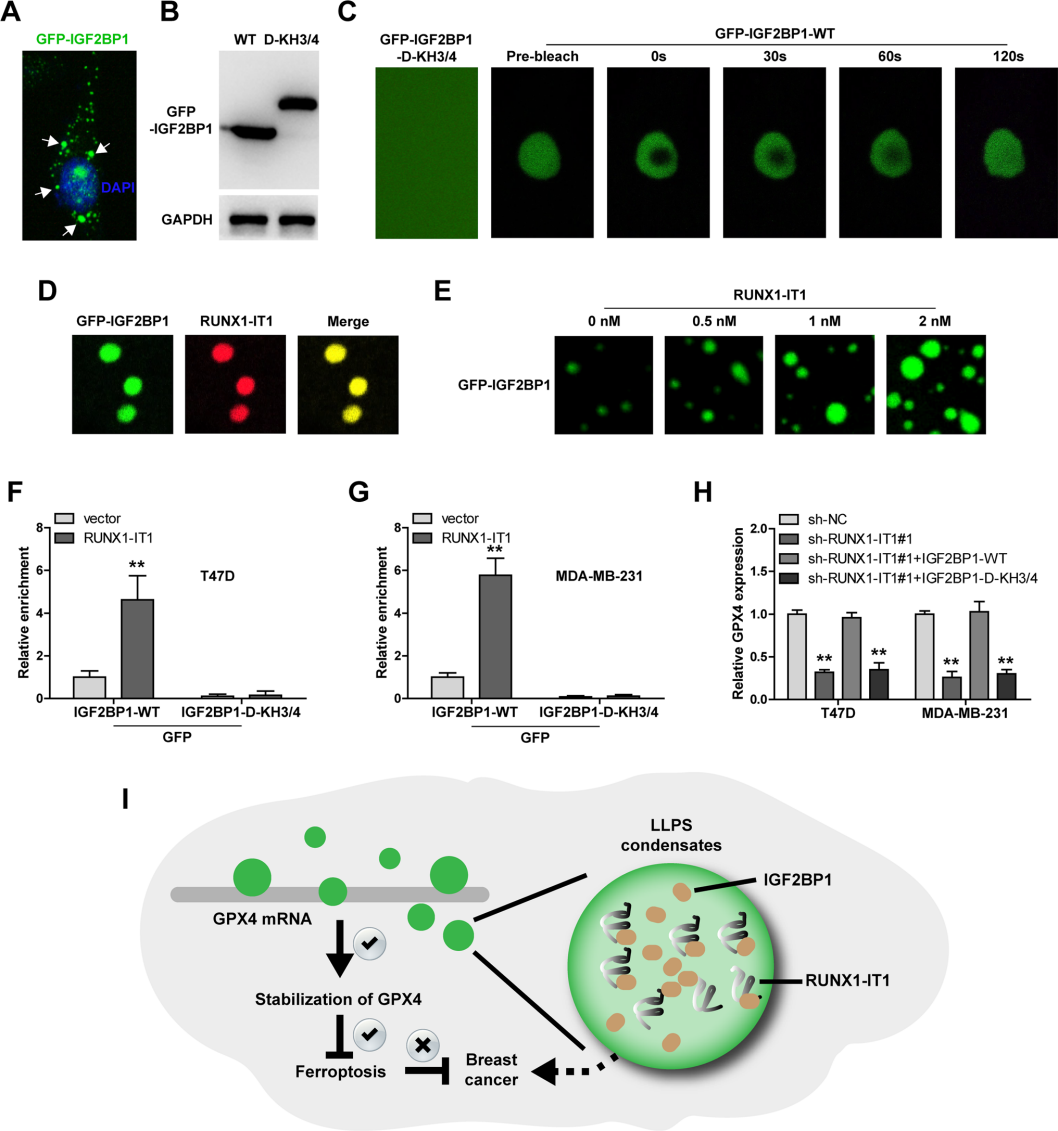
RUNX1-IT1 drives IGF2BP1 protein LLPS. **A** Detection of green fluorescent signal in cells transfected with GFP-IGF2BP1 vector. **B** Western blot verifying protein purification of IGF2BP1 and its mutant. **C** FRAP assay testing the fluxility of GFP-IGF2BP1 protein. **D**, **E** In vitro phase separation assay using GFP-IGF2BP1 protein and RUNX1-IT1. **F**, **G** RIP assay testing the binding of IGF2BP1 and its mutant to GPX4 mRNA in RUNX1-IT1-overexpressed cells. **H** qRT-PCR analysis of GPX4 mRNA expression in RUNX1-IT1-silenced cells transfected with IGF2BP1 and its mutant vectors. **I** The proposed model of RUNX1-IT1 promoting breast cancer by regulating GPX4-mediated ferroptosis via driving IGF2BP1 protein LLPS

Recent evidence shows that IGF2BP1 can form LLPS condensates critical for its function, and the KH3/4 domain of IGF2BP1 is required for LLPS formation [24]. As expected, discrete puncta was observed in cells transfected with GFP-IGF2BP1 vector (Fig. 6A). Then, IGF2BP1 and its mutant (KH3/4 domain deletion) were purified (Fig. 6B), followed by FRAP assay in vitro. The results showed that IGF2BP1, but not its mutant, formed liquid droplets, and underwent a highly dynamic fusion (Fig. 6C). Moreover, we mixed IGF2BP1 and RUNX1-IT1, and found that they were co-located in the liquid droplets (Fig. 6D), and RUNX1-IT1 increased the size and number of IGF2BP1 liquid droplets in a dose-dependent manner (Fig. 6E). Further, deletion of KH3/4 domain resulted in the loss of IGF2BP1’s ability to bind to GPX4 mRNA, even in the case of RUNX1-IT1 overexpression (Fig. 6F, G). And the reduced GPX4 expression caused by RUNX1-IT1 knockdown was rescued by overexpression of IGF2BP1 rather than its mutant with KH3/4 domain deletion (Fig. 6H). These data indicate that RUNX1-IT1 elevates GPX4 via promoting IGF2BP1 LLPS.

## Discussion

Extensive evidence suggests that lncRNA is a fundamental player in human cancer [25]. However, its role in breast cancer has not been characterized. In this study, we found that RUNX1-IT1 was overexpressed in breast cancer tissues in GEO, TCGA and our cohort. Inhibition of RUNX1-IT1 repressed breast cancer cell proliferation and invasion in vitro, and tumor growth in vivo. Deep mechanism investigation revealed that RUNX1-IT1 directly bound to IGF2BP1 and promoted LLPS condensate formation of IGF2BP1, leading to increasing binding of IGF2BP1 to GPX4 mRNA, inhibiting GPX4 mRNA decay (Fig. 6I). As a result, the elevated GPX4 blocked lipid peroxidation and ferroptosis, thereby facilitating breast cancer tumorigenesis (Fig. 6I). Therefore, our study suggests that RUNX1-IT1 is a novel oncogenic lncRNA in breast cancer, dysregulation of RUNX1-IT1/IGF2BP1/GPX4 is responsible for breast cancer carcinogenesis and progression.

The mechanism of action of lncRNA is extremely complex and has not been fully understood. Emerging evidence shows that lncRNA regulates gene expression via serving as a protein binding partner [26]. Deleted in lymphocytic leukemia 2 (DLEU2) was able to directly interact with Hepatitis B protein and enhancer of zeste 2 polycomb repressive complex 2 subunit (EZH2), leading to transcriptional activation of a subset of genes related to hepatocellular carcinoma [27]. LncRNA uc.291 functioned as a suppressor in cutaneous squamous cell carcinoma via binding to chromatin remodeling complex SWI/SNF [28]. Gastric cancer associated transcript 2 (GACAT2) could bind to pyruvate kinase M1/2 (PKM1/2), and was identified as a regulator in cell mitochondrial function and inflammatory environment [29]. Herein, we found that RUNX1-IT1 was capable of binding to IGF2BP1 protein, a stabilizer of mRNA via recognition of m^6^A motifs [30]. Silencing of IGF2BP1 blocked the increased GPX4 expression caused by RUNX1-IT1 overexpression, hinting that IGF2BP1 is required for RUNX1-IT1-mediated regulation of GPX4. Further research is needed to determine how IGF2BP1 and RUNX1-IT1 interact with each other and their specific binding regions, so as to design some oligonucleotides or peptides to block their binding, which may be a novel therapeutic strategy for breast cancer.

Cells are separated by many membrane-closed organelles and non-membranous compartments to ensure that a variety of cell activities occur in a spatiotemporal controlled manner. Molecular mechanisms of membrane-bound organelle dynamics, such as their fusion and fission, vesicle-mediated transport and cell-cell interaction mediated by membrane contact, have been widely characterized. However, the molecular details of the assembly and function of the membraneless compartment are still elusive [31]. Recent evidence shows that many non-membrane compartments have been assembled through LLPS, which are collectively referred to as biomacromolecule condensates [32]. Formation of LLPS condensates is critical for the function and activity of biomacromolecules, such as proteins and nucleic acids [33]. IGF2BP1 protein is a m^6^A reader that has been shown to be able to form LLPS condensates under physiological conditions, and the KH3/4 domain is required for LLPS formation [24]. Here, we verified that IGF2BP1 polymerized into droplet-like condensates both in vitro and in vivo. Deletion of KH3/4 domain led to IGF2BP1 condensate disappearance, as well as the loss of the binding of IGF2BP1 to GPX4 mRNA, suggesting that IGF2BP1 LLPS is key for regulation of GPX4 mRNA stability. More strikingly, we found that RUNX1-IT1 was co-located with IGF2BP1 in the liquid droplets, and RUNX1-IT1 promoted IGF2BP1 LLPS formation in a dose-dependent manner, which can be explained by the notion that RNA regulates protein LLPS via direct binding [34]. How RUNX1-IT1/IGF2BP1 liquid droplets are formed, and whether there are other key proteins or nucleic acids in these droplets, still need further study.

This study has some limitations, the major weakness is that the breast cancer samples used in our research are retrospective, which may bias the conclusions. In addition, the patient-derived tumor xenograft (PDX), a model closest to the actual situation of the human body, was not used in this study, which may diminish the clinical application value and prospect of our conclusions.

Collectively, our data reveal that RUNX1-IT1 promotes breast cancer tumorigenesis through inhibiting ferroptosis via regulating IGF2BP1/GPX4 axis, providing a potential prognostic marker and therapeutic target for breast cancer.

## Supplementary Information

Supplementary Material 1

## Data Availability

The datasets used and/or analyzed during the current study are available from the corresponding author on reasonable request.
