# Supplementary material for: RUNX1-IT1 favors breast cancer carcinogenesis through regulation of IGF2BP1/GPX4 axis
Source: Discov Oncol. 2023 Apr 10;14:42. doi: 10.1007/s12672-023-00652-z (PMC10086083; doi:10.1007/s12672-023-00652-z)
Supplement: Supplementary file 1 — Supplementary Material 1 [file 12672_2023_652_MOESM1_ESM.doc]

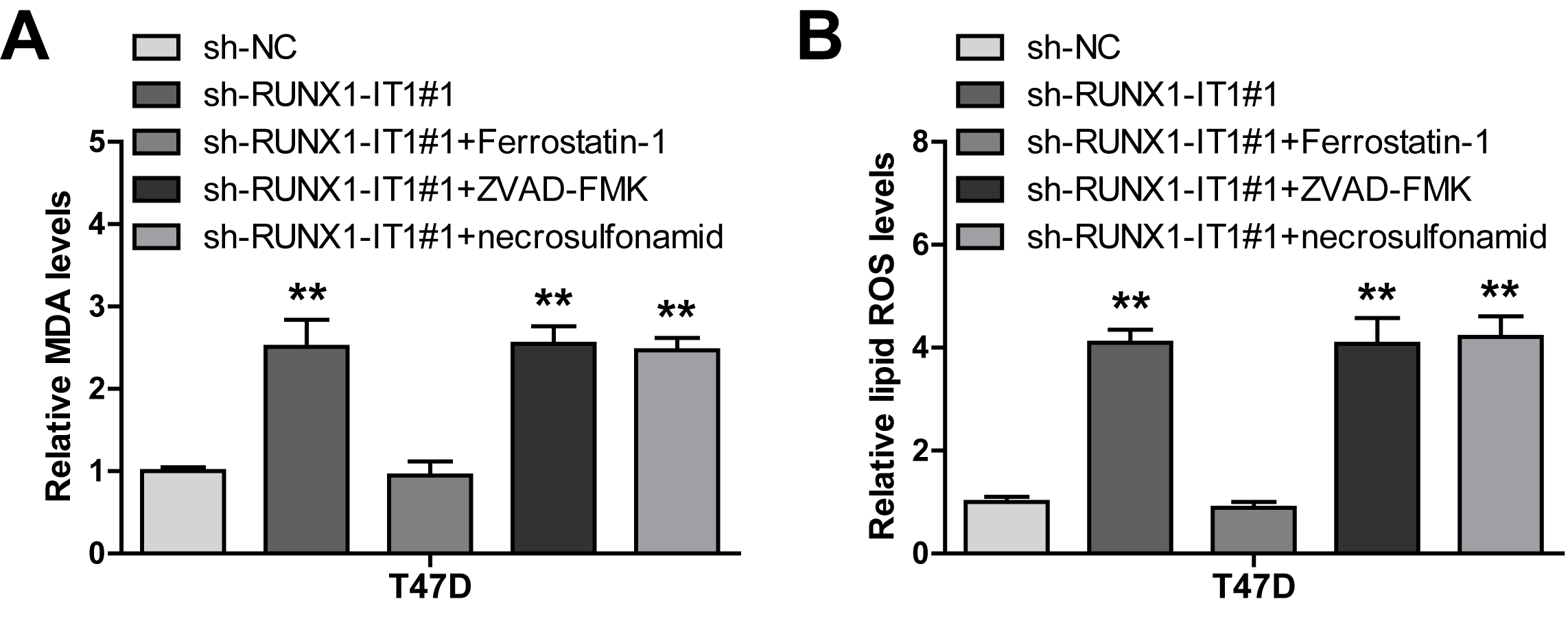


**Figure S1.** Detection of MDA and lipid ROS levels in RUNX1-IT1-silenced T47D cells treated with 1 µM Ferrostatin-1, 10 µM ZVAD-FMK or 0.5 µM necrosulfonamide.

**
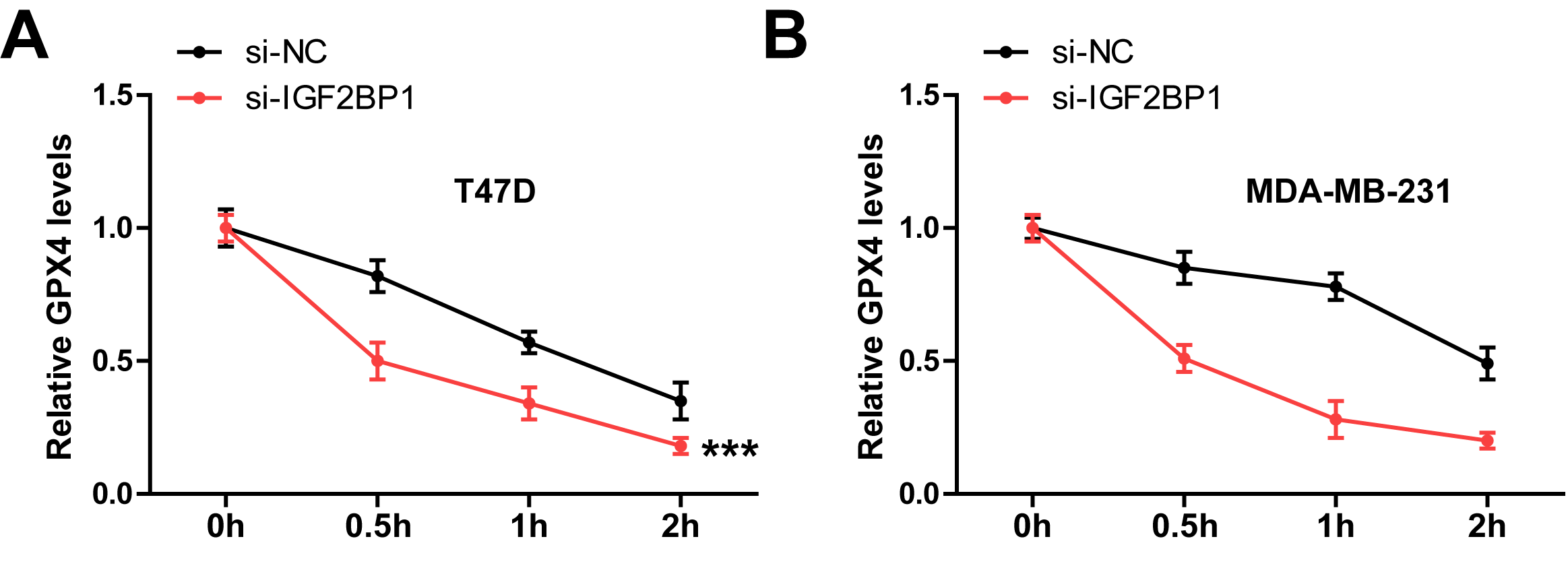
**

**Figure S2.** qRT-PCR analysis testing the half-life of GPX4 mRNA in IGF2BP1-silenced cells treated with 10μM actinomycin D at the indicated time.
